# Dialogued into being: Constructing knowledge about hand osteoarthritis from a polyphony of voices in healthcare encounters

**DOI:** 10.1080/17482631.2024.2330221

**Published:** 2024-03-18

**Authors:** Hege Johanne Magnussen, Ingvild Kjeken, Irma Pinxsterhuis, Trine Amalie Sjøvold, Marte Feiring

**Affiliations:** a Department of Rehabilitation Science and Health Technology, Faculty of Health Sciences, Oslo Metropolitan University, Oslo, Norway; b Norwegian National Advisory Unit on Rehabilitation in Rheumatology, Diakonhjemmet Hospital, Oslo, Norway; c REMEDY - Centre for Treatment of Rheumatic and Musculoskeletal Diseases, Diakonhjemmet Hospital, Oslo, Norway; d REMEDY Patient Council, Diakonhjemmet Hospital, Oslo, Norway

**Keywords:** Reflexive thematic analysis, dialogue, clinical encounters, health professional knowledge, experiential knowledge, knowledge construction

## Abstract

**Purpose:**

Multiple knowledge sources inform healthcare. In healthcare encounters, patients and health professionals’ ideas intersect to understand illness and disease. Exploring what is thought of as legitimate knowledge, and where those reflections come from is central to the process of improving and developing healthcare. Within this context, we aim to explore how knowledge about hand osteoarthritis (OA) is constructed and negotiated in clinical consultations.

**Methodology:**

The article is based on interviews with 21 patients and 14 health professionals in combination with observation in 16 clinical consultations. Reflexive thematic analysis was used to interpret the data.

**Results:**

We generated four themes from codes to tell an interpretive story about how hand OA meaning-making is “talked into being” in patient-provider encounters: from the dominant voice of health professionals, from patients as knowers in the chronic healthcare dialogue, from health professionals and patients constructing knowledge together and from the construction of knowledge in hybrid positions when patients are health professionals and health professionals have hand OA.

**Conclusion:**

New knowledge about hand OA is co-constructed in the situated context of the clinical encounter through a polyphony of voices—some of which are dominant, while others occupy the periphery—within and between the interactants in dialgue.

## Introduction

Healthcare is a system of expertise and is based on exclusive health professional practices (Palukka et al., 2021; Thorne, 2008). In healthcare encounters, health professionals control a type of knowledge beyond experiences from everyday life (Freidson, 2001; Parsons, 1951), and their work is legitimized by the idea that health professionals have expert knowledge within health and medicine (Abbott, 1988; Grimen, 2009). This system has abstract power to define and solve problems (Abbott, 1988), and is sourced from a synergy of theoretical and practical skills that are required to address and deal with disease (Grimen, 2009).

Simultaneously, it is widely acknowledged that clinical status and the diagnosis cannot solely define and solve health problems (Mol, 2008). Instead, patients’ meanings and experiences of illness are increasingly recognized as ways of knowing that are different to those of health professionals with their societal and institutional authority (Baillergeau & Duyvendak, 2016; Halloy et al., 2023). The shift from labelling patient experiences as lay beliefs to characterizing them as lay knowledge and expertise contributes to an enhanced acceptance of experiential knowledge as being valuable in healthcare (Blume, 2017; Greenhalgh, 2009; Prior, 2003). Knowledge in healthcare can therefore be understood as a relational, collaborative achievement between professional and patient in partnership, in what Rose and Kalathil (2019) denote the promised third space of co-production. At the same time, researchers have pointed to significant friction between the making of experiential patient claims and the scientific claims from established biomedical knowledge (Mahr, 2021), where the lack of trustworthiness assigned to patient accounts in various health settings corroborates epistemic injustice (Fricker, 2007). Within such a context, knowledge democratization remains of secondary importance to the elite academic production of knowledge for healthcare (Ferlie et al., 2012). In consequence, power asymmetries are sustained, with health professionals in the more dominant positions (Dent & Pahor, 2015).

In institutional healthcare settings, knowledge about health and illness is negotiated at the intersection between the professional knowledge of the health providers and the experiential knowledge of patients. Whilst health professionals’ voices often orient to the general, objective knowledge and professional experiences in their specialized health field, patients voice particular and subjective symptom experiences in a more holistic and total manner that is oriented to the here and now (Borkman, 1976; Castro et al., 2019; Lindström & Karlsson, 2016). This subjectivity is not only associated with individual characteristics, but also with situations and interactions. To talk about patients’ and health professionals’ different positions rather than perspectives is therefore more applicable when aiming to understand the construction of knowledge in healthcare (Pols, 2005).

In this article, we explore how knowledge about health and illness is constructed and negotiated in healthcare interactions through Bakhtin’s (1981, 1984, 1986) ideas about polyphony and dialogue. We aim to focus attention on how meaning is produced relationally by patients and health professionals as they position themselves towards, and respond to, each other in consultations. Bakhtin (1984) who was a philosopher and literary critic used the term polyphony when describing the complexity of the characters in Dostoevsky’s novels. Polyphony entails the coming together of the plurality of viewpoints that are present in human dialogue and the creation of new understandings. We interpret Bakhtin’s polyphony perspective to coincide with the idea that, rather than viewing healthcare as a process of transferring knowledge from health professionals to patients, patients are involved in a dialogue that aims to search for understanding: *“Truth is not born nor is it to be found inside the head of an individual person, it is born between people collectively searching for truth, in the process of their dialogic interaction”* (Bakhtin, 1984, p. 110). Central to Bakhtin’s work is the process of dialogue as unfinalized and dynamic, where the actors position themselves in relation to others through a polyphony of past and present voices. Through the lens of Bakhtin, we explore how patients and health professionals construct knowledge about health, illness, and disease in dialogue with, and by positioning themselves towards, the worldviews of others. Bakhtin (1981) points to the tension between forces towards unity (centripetal) from own perspectives and the forces towards difference (centrifugal) that come into existence through the ideas of others. When such compatible or conflicting accounts of reality intertwine in sense-making through dialogue as a joint achievement (Gergen, 2015), complex interpretations and negotiations are generated in the act of understanding.

Bakhtin’s theory of polyphony and dialogue has been widely used beyond linguistics and literary studies. His ideas are utilized in organization studies (Belova et al., 2008; Hazen, 1993) and in the study of healthcare organizations (Sullivan & McCarthy, 2008). His framework is also employed in studies about navigating health transitions (Renedo et al., 2020), boundary positions of doctor-managers (Iedema et al., 2004), construction of patient-centredness between nurses and patients with chronic illnesses (Phillips & Scheffmann-Petersen, 2020), ways of knowing in nursing (Bowers & Moore, 1997) and the construction of health professional knowledge (Renedo et al., 2017). We find Bakhtin’s perspectives especially suited to exploring how a polyphony of voices interact in clinical encounters. In a dialogical understanding of knowledge in healthcare, we emphasize that patients and health professionals contribute with a multitude of health, illness, and disease positions to inform healthcare (Kleinman, 1980) when they construct and share new meaning together (Gergen, 2015).

By referring to patients rather than persons in this article, we run the risk of undermining the holistic thinking that underpins person-centredness (Håkansson Eklund et al., 2019; McCormack, 2003), which we support. Our decision stems from the voices of our study participants, who commonly talked about patients instead of persons with hand OA, which might reflect the continued dominance of patient over person in Norwegian healthcare. Our intention is not to highlight health professionals’ expertise by using the word patient. On the contrary, we stress the importance of particularizing the person that the patient is, beyond just the healthcare perspective (Fleming & Mattingly, 2019).

We see healthcare consultations as purposed and structured social events (Heritage & Maynard, 2006), and we agree with Mol (2008) who argues that healthcare must align with the unpredictable reality of everyday life rather than be understood as a series of decisions made by patients or clinicians at particular times. By using Bakhtin’s ideas about polyphony and dialogue, we aim to understand knowledge in healthcare beyond individualist and opposing views of knowledge as formed either by health professionals or patients. Rather, we understand knowledge construction as a dialogical process where patients and health professionals build action in concert with each other (Gergen, 1994a). In that interplay, their voices form a multivocal and often contradictory unity when multiple sources of knowledge are increasingly being recognized as legitimate in healthcare. Understanding what is considered legitimate knowledge about health, illness, and disease, and how those truths come about, are fundamental aspects that need to be explored in optimizing and further developing healthcare. In efforts to contribute to such an understanding, we explore how knowledge about a chronic condition of the hands is constructed and negotiated when patients and health professionals interact in hand osteoarthritis (OA) healthcare consultations.

## Methodology

### Study context

Hand OA is described as a heterogenous condition where both symptom experiences and illness course differ between patients (Kloppenburg & Kwok, 2012). The condition affects one in two women and one in four men (Qin et al., 2017). The chronic manifestations often described in hand OA begin clinically silently, manifest over years, and cause pain, stiffness, and loss of function. Hand OA can be classified according to radiographic results, symptoms or clinical features (Favero et al., 2022). Key interventions as recommended by The European League Against Rheumatism are patient education, exercises for the hand and the use of assistive devices (Kloppenburg et al., 2018). Contemporary ideas about and practices regarding hand OA are constructed within wider sociopolitical contexts. In Norway, hand OA should mainly be attended to in primary healthcare (Norwegian Directorate of Health, 2015). However, relevant service availability is limited, resulting in referrals to specialist care. The strong association with ageing and the lack of a cure contribute to the ranking of hand OA at the lower end of the disease prestige hierarchy (Album & Westin, 2008), which has consequences for the understanding and positioning of hand OA in modern healthcare.

### Recruitment

The study was approved by The Regional Committee for Medical and Health Research Ethics (case number 2017/742, 2020/8450) and the Norwegian Centre for Research Data (reference number 197320) and was conducted in line with the Declaration of Helsinki. We recruited participants from December 2020 to February 2023 as part of a larger research project on task-shifting in hand OA care. The larger research project consists of a completed, randomized controlled trial in two hospitals specializing in rheumatology (Kjeken et al., 2021), ongoing qualitative studies in four hospitals and a forthcoming Delphi consensus exercise in 2024. In our study, twenty-three patients and 15 health professionals were initially contacted by local study coordinators in two hospitals. Those who agreed to participate were subsequently contacted by the first author (HJM) for further information about the study aim, participant confidentiality and the informed consent process, including possible withdrawal from the study at any time without consequences. Two patients and one health professional declined participation, stating illness and lack of time as causes, giving a total of 35 interview participants. Table I summarizes their demographic characteristics.

**Table I.** Summary participant demographics.

	Occupational therapist (8)	Rheumatologist (6)	Patients participating in interviews (21)	Patients participating in observations (16)
*Gender*				
Male	1	1	6	5
Female	7	5	15	11
*Age*				
Under 40	4	1	–	–
40–55	1	4	1	6
Over 55	3	1	20	10
*Years of professional and/or lay experience with hand OA*				
Under 2	3	–	–	3
2–20	2	5	21	8
Over 20	3	1	–	1
Not known	–	–	–	4

During interviews with health professionals, HJM asked for permission to observe clinical hand OA consultations, upon approval from patients. Seven health professionals agreed while the other seven had changed jobs or said that they no longer performed consultations with hand OA patients. We were not able to observe the patients we had already interviewed, as they no longer consulted healthcare for their hand OA. New patients were therefore approached by study coordinators for inclusion based on identification of relevant consultations. HJM had established a relationship with health professionals prior to participating in 16 consultations when meeting the new patients for the first time. Prior to interviews and consultations, written informed consent was obtained from participants. In viewing ethical judgement as relational and contextual rather than prescriptive (Riessman & Mattingly, 2005), we continuously negotiated informed consent (Klykken, 2021) when the conversation took new and unexpected directions. In situations where participants lowered their tone of voice or looked extensively at their hands rather than maintain eye contact, they were asked if they wanted to turn off the dictaphone, pause, or end the interview. We did this in efforts to be consistent in viewing informed consent as an ongoing process, and to maintain stronger rather than weaker consent as weak consent generates poorer data (Miles & Huberman, 1994).

### Reflexivity

As a researcher, reflexivity is central when performing reflexive thematic analysis (Braun & Clarke, 2021). Each of the research team members was part of the working process throughout, bringing various ideas to the study. We engaged continuously in an iterative process, where we strived to move between standpoints and reflect on our own perspectives, positions, and activities, and how we influenced the data. Our research team included a patient research partner with extensive familiarity of living with hand OA (Trine Amalie Sjøvold), and a mix of academics. Ingvild Kjeken is a professor and occupational therapist with longstanding research/clinical experiences in rheumatology. Irma Pinxsterhuis is an associate professor and occupational therapist. Marte Feiring is a professor, sociologist, and occupational therapist. Hege Johanne Magnussen is a PhD student and a physiotherapist. While the latter three were less well-acquainted with rheumatology and hand OA at the start of the project, they each brought experiences from years of working with chronic illness and disability into the research process.

### Data generation

We approached our research question through interviews and observations. Interviews generated data about what participants said about their views and actions in consultations. Data from observations gave us access to the actual actions of participants in clinical encounters. Interview guides were initially developed and piloted (Supplementary material 1) before HJM conducted all interviews. Interviews lasted for 45–90 minutes. Five were conducted digitally and 30 took place face-to-face, according to participants’ preferences. Interviews were conducted in a dialogical style where efforts were made to highlight the significance of participants’ various viewpoints to the study. Interviews were audio-recorded and transcribed verbatim by HJM, except from three interviews that were transcribed by a research assistant.

HJM subsequently negotiated interactive observation in 16 clinical consultations (Wind, 2008) that were 30–60 minutes in length, thereby gaining an insider perspective through participation while also being placed on the outside as an observer (Spradley, 1980). During data generation, as HJM is also a health professional, there was an emphasis on voicing the PhD student position, underlining the non-affiliation with the two hospitals, and forwarding clinical requests during interviews to study coordinators. In addition, participants were approached as experts in sharing their ideas.

We used Malterud et al. (2016) information power concept to evaluate the quality and richness of the generated data where the study objective, participant specificity, theoretical framework, quality of interviewing and analytical approach are key components in informing the decision to halt data generation. After multiple research team discussions, we evaluated that information power was met after 35 interviews and 16 observations. Our evaluation was based on the use of established theory, HJM’s experiences with qualitative interviewing from previous qualitative research, and the depth and breadth of information gathered from our three-year engagement with the data. Table II summarizes data generation. The secure platform Services for Sensitive Data was used to store data in compliance with the Norwegian privacy regulation. Audio files were directly encrypted and transferred post-interview. Interview transcripts and field notes were thereafter uploaded to NVivo to aid the analytical process.

**Table II.** Data generation summary.

Data source	Hospital A	Hospital B
Interviews with patients	9	12
Interviews with health professionals	Rheumatologists (3) Occupational therapists (3)	Rheumatologists (3) Occupational therapists (5)
Observation in clinical consultations	Clinical consultations rheumatologists and persons with hand OA (0)	Clinical consultations rheumatologists and persons with hand OA (5)
	Clinical consultations occupational therapists and persons with hand OA (4)	Clinical consultations occupational therapists and persons with hand OA (7)

### Data analysis

Data were analysed using reflexive thematic analysis (Braun & Clarke, 2006, 2019, 2022b) from a constructionist frame of reference, where we focused on socially produced ideas and assumptions about hand OA rather than participants’ individual attitudes and values in our engagement with the data (Braun & Clarke, 2022a). By combining different perspectives and actions from various knowledge communities, we aimed to strengthen our understanding of the interactional dynamics in play when knowledge about health, illness and disease was presented and acted upon in clinical practice. In doing so, we pay attention to the mutual constitution of meaning between participants, researchers, the research context and the larger context (Braun & Clarke, 2022b).

We were already familiar with the content of the data from prior analysis of two separate data sets: transcripts from patient interviews that resulted in one article (Magnussen, Kjeken, Pinxsterhuis, Sjøvold, Hennig, et al., 2023), and transcripts from health professional interviews combined with field notes from observations that resulted in a second article (Magnussen, Kjeken, Pinxsterhuis, Sjøvold, & Feiring, 2023). Guided by our research question about how knowledge is constructed in clinical encounters, HJM made efforts to analyse this combined dataset from a new angle, focusing on new data that had not been explored and presented in the two previous articles while also acknowledging that those previously developed analysis influenced the present process. After a thorough re-familiarization with the interview transcripts and field notes, involving reading and re-reading while also taking reflective notes and recording salient ideas, HJM engaged in an open and organic coding process that was guided by the research question. Aside from the manifest codes initially developed inductively, latent codes were subsequently created in a process influenced by theory of power (Lukes, 1974), knowledge (Gibbons, 1994; Nowotny et al., 2003) and negotiations (Strauss, 1963) as codes were refined and developed into preliminary themes. Themes were subsequently discussed, refined, re-discussed, and further distilled by the research team. This abductive activity (Alvesson & Sköldberg, 2018) at the intersection between the data, theoretical interests and the larger context resulted in the development of patterned meaning across the dataset to tell an overarching interpretive story (Braun & Clarke, 2022c) (Table III).

**Table III.** Example of the analytical process from familiarization to codes to themes development.

**Examples familiarization notes**			
Diverse explanatory models of health, illness and disease intersect in the construction of knowledge about hand OA. Knowledge about hand OA is generated through conversations with others in similar situations. Past experiences direct the understanding of hand OA in current consultations. Patients and health professionals complement each other in understanding hand OA. Uneven relationship when patients navigate unfamiliar terrain while health professionals operate on their own turf of the hospital. Health professionals and patients generate solutions to patient problems together. Chronic illness creates room for stronger relevance of patient experiences. The acquisition of new knowledge from science makes changes to clinical practice. Health professionals in specialist healthcare have the valid knowledge about hand OA. Osteoarthrosis a common condition where dual positions from being both health professionals and patients occur. Participants who are patients talk about how little they know about hand OA despite living with it over many years. Answers to patient problems found within the healthcare system, not with patients themselves. Patients often confirm health professional information: either health professionals are really good in exploring exactly what patients need or maybe patients see it as a way of contributing to a good relationship. To be polite? Create a good atmosphere? Test health professional knowledge? Do what is expected? Desire to learn more? The lack of a cure limits the value of biomedicine and contributes to balance knowledge differences.			
**Examples of codes**			
Patients depend on HP knowledge to solve problems Patients are passive recipients of hand OA knowledge Patients adapt to health professional views HPs control time, space, content, and transitions in clinical encounters Taken for granted hierarchy of ideas from history and culture HPs powerful position warrants voice HPs are experts by evidence HPs use scientific claims to legitimize own viewpoints Outdated patient ideas regarding hand OA Negotiation space limited when HPs know best Hand OA knowledge construction a linear process	Accrued lay understandings of hand OA brought into meaning making in consultations Patients claim no-knowledge about hand OA Experience from living with hand OA not epistemically relevant HPs view experience as valid knowledge Patient experiences actively used to understand hand OA Chronic condition with no cure call for recognition of experience	Complementary exchange of illness and disease ideas to jointly make sense of hand OA Discrepancy in illness and disease understandings create new knowledge Confirmation of subjective illness experiences facilitate more active patients in consultations Patient experiences trumps international recommendations in rheumatology Pain as both harmful and harmless Activities as both restricted and necessary Professional uncertainty an added value in the consultation dialogue HPs include patients in the clinical reasoning process Knowledge as relational Knowledge co-developed in local contexts Interactants participate from more equal positions	The patient HP navigates familiar territory Health professional background an added value for patients in understanding hand OA The patient HP act in line with expectations Patients do not disclose own HP background HPs openly communicate hand OA status to patients HPs with hand OA use own experiential knowledge in consultations Understanding hand OA from the other side Interactants with similar experiences enhance dialogue
**Preliminary themes from codes**			
Knowledge construction a monolog from HPs to patients	Patients as experts by experience warrants central position in dialogue	Dialogue of equals in local contexts	Constructing knowledge from dual positions
**Themes**			
*Theme 1* HPs dominate dialogue when they know best	*Theme 2* Patients are knowers in the chronic healthcare dialogue	*Theme 3* HPs and patients construct knowledge together	*Theme 4* Dialogue takes place from double positions when patients are health professionals and health professionals have hand OA
**Overarching interpretive story from the themes**			
Hand OA sensemaking is dialogued into being in local patient-provider encounters			

**Figure 1.**
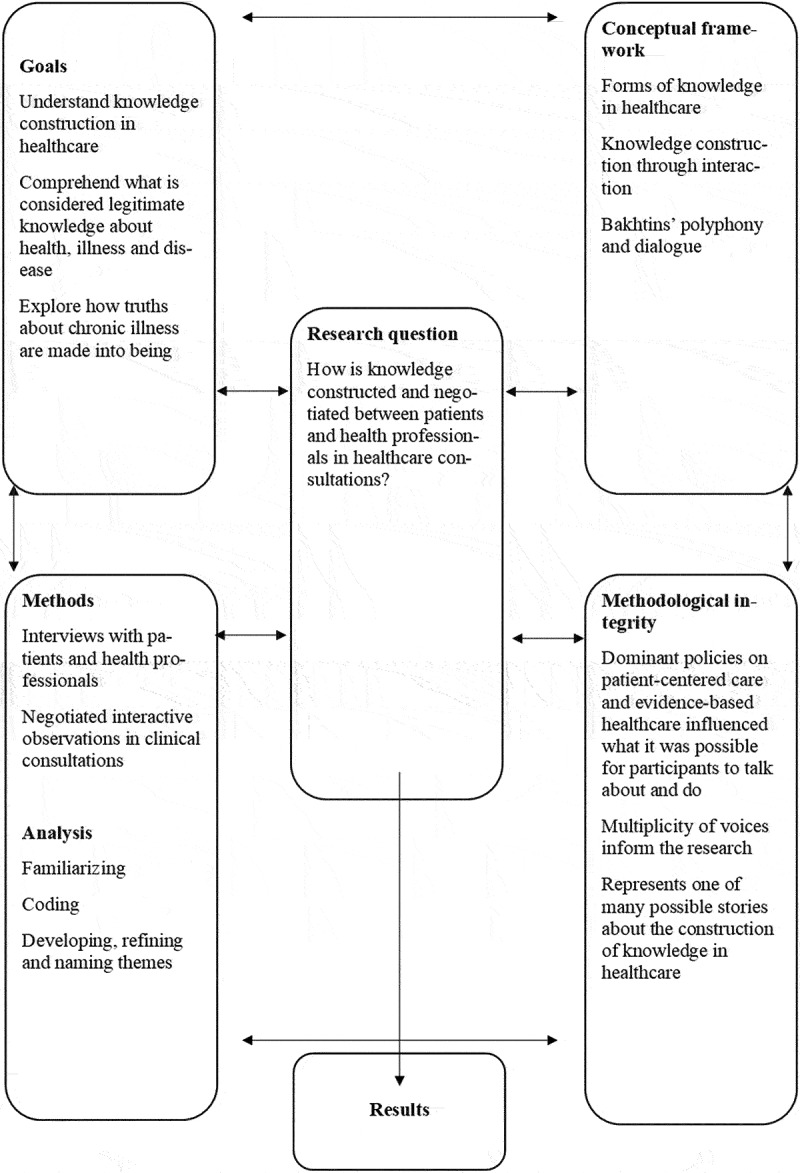
Research design map using Maxwell’s (2013) interactive model for qualitative research design.

We strove to encourage dialogue throughout the data generation and analytical process, where we used reflexive openness to welcome differing viewpoints and to make sense of these multiple ideas in context. Combining interviews and observations to generate data, prolonged engagement with the field, deep and insightful interactions with the data set, and describing the analytical process in detail were approaches we developed to strengthen the rigour of the study. A research design map is presented in Figure 1.

## Results

Data from our study generated four themes from codes about the construction and negotiation of hand OA in consultations: from the dominant voice of health professionals in dialogue, from patients as knowers in the chronic healthcare dialogue, from health professionals and patients constructing knowledge together, and from hybrid positions when patients are health professionals and health professionals have hand OA.

### The dominant voice of health professionals in dialogue

Our first theme was centred on the construction and negotiation of knowledge about hand OA from dominant health professional positions. Hierarchical distance between patients and providers influenced interactions where professional knowledge was most valued. In interviews, patients talked about health professionals’ knowledge as something patients depended on: “*I’m probably of that type and age where I think that they [health professionals] are the ones who should know, not me. When the doctor has said it, then it must be true*” (P15). From such a position, knowledge about hand OA was understood as an entity that health professionals had and made active use of *for* patients at the receiving end. In these encounters between active professionals and passive patients with hand OA, the patients often adopted the views of health professionals rather than dynamically engaging in the construction of new knowledge. Health professionals in our study referred to similar situations: “*If the patient is affected by this and it influences their ADL [activities of daily life] and their life situation […] because they don’t want to be in pain, they want to function, then they’ll take everything you offer—everything you recommend, they’ll take it all*” (R2). Health professionals also emphasized their own expertise about hand OA: “*It really helps a lot to know that I’ve worked so considerably with this [hand OA] and seen so many patients, right, and know a lot, and that I can be confident and know that what I’m saying is correct, and yes, I think that means something, that we can convey such confidence regarding us having knowledge about this [hand OA]*” (OT1), and how that expertise was enacted in consultations: “*So basically, just confirm the thoughts they already have as long as they are correct. Or refute them if they’re not*” (OT6). Patients were expected to have an underlying commitment and to adapt to provider knowledge: “*This is a patient who’s going to do what you say*” (OT1). In such encounters, knowledge about hand OA was constructed from the dominant voices of health professionals, with limited space for negotiations, and transferred on to unknowing patients whose voices occupied the dialogue periphery.

Health professional dominance was also visible in observations. Their speech and body language guided patients to enter the consultation room, sit in a designated chair, lay on a bench, look at screens and sketches, and respond to the health professional’s questions as they unfolded. The provider control over consultation time, space, content, and transitions in clinical encounters showed how health professionals’ understandings from historical practices became strong voices that influenced current interactions. Health professionals in our study often initiated consultations by asking patients if they knew what hand OA was, followed by a proposal to provide relevant information. Without exception, patients accepted the information offered, including those patients who claimed to have previous knowledge about hand OA. Health professionals talked about the challenges of continuous engagement with emerging research while also using scientific claims to legitimize the information they provided to patients: “*You have to be able to document why you do what you do, and often that comes down to research*” (E6). In highlighting science, knowledge about hand OA was monologically transferred from health professionals to patients, rather than being co-constructed between them in dialogue, and health professionals demarcated their own scientific voices from the non-scientific voices of patients (Gieryn, 1983). This shows the centrality of health professionals’ expert knowledge in the polyphony of voices that govern patient-provider interactions.

In our study, patients often described hand OA as being caused by wear and tear. In contrast, health professionals talked about wear and tear as an outdated way of understanding the cause of hand OA. Thus, health professionals positioned their own epistemic resources as up to date and central in the dialogue when multiple understandings were interweaved. By imposing their own knowledge of hand OA on the patients within a technical framework instead of starting a conversation, the subsumed social knowledge of the patient was left in the periphery. Our analysis shows how dominant health professional voices influence the consultation dialogue when providers posit knowledge that un-knowing patients need. In such encounters, patients and providers represent two distinct voices that respond to each other from unequal positions of influence. This linear construction of knowledge starts with patients presenting their problems and ends with health professionals solving those problems. Such a dialogue is framed within an implicit hierarchy of ideas, where powerful health professional positions justify a principal voice (Burr, 1995). Patient voices in the periphery make it challenging to negate historical health-professional-knows-best practices.

When the strong orientation towards listening to health professionals’ ideas dominates the dialogue, the different voices of health professionals and patients are activated from various knowledge sources. These ideas co-exist in time, but with little joint meaning between interactants, and therefore contribute to preserving particular health professional interests. At the same time, centripetal forces towards harmony are also active in such encounters when health professionals are considered by both patients and health professionals to have legitimate knowledge about hand OA.

### Patients are knowers in the chronic illness dialogue

Our second theme captured how knowledge about hand OA was constructed and negotiated from patients’ experiences when patients in our study brought personal accounts of living with a chronic condition not known to health professionals into consultations. Patients described how they discussed illness experiences with family, colleagues and friends, thereby negotiating understanding about hand OA from outside of the clinical environment (Kleinman, 1980). The polyphony of voices from patients’ everyday lives made them reflect and interact not only from individual experiences but also from a collection of lay viewpoints that they accrued and brought into the consultation. Through these multiple voices, patients positioned themselves as actors without knowledge about health and hand OA in the dialogical encounter with health professionals when they talked about how they “*know nothing about health*” (P21) and were “*not a scholar*” (P1), and that they’d “*never encountered this osteoarthritis before, so […] didn’t know anything*” (P3). By establishing that they did not know, patients’ own experiences from having hand OA were not considered as epistemically relevant in encounters with health professionals within the institutional framework of healthcare.

Contrary to such understandings of epistemic inferiority by patients, the health professionals in our study talked about patients as experiencing individuals rather than objects with hand OA, and turned patient experiences into epistemic resources by referring to patients as experts: “*They are the experts in how they feel and what they’re dealing with, and I have my knowledge and expertise in the field of osteoarthritis and know a lot about that […] those [patients] are the experts on their own lives and they are the ones who are important for improvement to take place*” (OT8). Health professionals further emphasized the importance of how patients experienced and understood hand OA: “*There can be similar findings on two people’s hands […] and then the experience of suffering can be very different, so I think it’s very, very relevant to get a complete picture and bring in the patient’s experiences and symptom burden, and yes, the whole problematic […] so it’s a really important part of the consultation. Not just what I see on the ultrasound, but the patient’s history, medical history, and symptoms. That’s what matters most—almost everything*” (R3). Lay experiences were given a central position in the dialogue by health professionals when they “*are guided by the user experience all along, that’s absolutely essential all the time*” (OT5) in clinical practice. This illustrates how patient experiences were pivotal when blending with the more peripheral voices of health professionals to construct knowledge about hand OA, using patient experiences as a vital resource in the creation of knowledge about hand OA.

The observation of a rheumatologist and his patient in a clinical encounter further illustrated how health professionals included patient experiences in the construction of knowledge about hand OA. The rheumatologist actively involved the patient in a collaborative quality control exercise for the information in the patients’ journal. By including the patient and instigating a joint evaluation of the medical journal history, the experiences of patients were actively brought into an understanding of hand OA. A polyphony of professional voices from the past and the present were combined with patient experiences in the process of hand OA meaning making. The amalgamation of voices from the rheumatologist, the patient journal and the patient created common understandings and was transformed into shared content for the patient to comment and elaborate on, together with the health professional. Discrepancies between the information available in the patient journal and a patient’s experience would often cause laughter in interactions, which positively influenced the consultation dynamics, silenced medical voices from the past, and enhanced the centrality of patient experience as knowledge in the current dialogue.

### Patients and health professionals construct knowledge together in dialogue

Our third theme centred on the creation of shared meaning from a multitude of resources that patients and providers brought into the encounter in constructing knowledge as an act of togetherness (Burr, 2015). In our study, patients’ experiences did not always correspond with the biomedical understanding of hand OA, thereby illustrating the multiplicity of illness and disease meanings in patient-provider dialogue (Hofmann, 2016; Mol, 2002). The lack of consistency between patients’ experiences of hand pain and the detection of few signs of OA in the hands by diagnostic x-rays and ultrasound is well-known (Glickel, 2001) and was also a common occurrence in our study. In such situations, health professionals acknowledged the subjective experiences of patients’ pain despite inconsistencies with the powerful voices of medical imagery in diagnosing disease. Therefore, patient experiences were positioned closer to the centre of the knowledge construction dialogue in shaping how hand OA manifested and was managed. Although central to disease understanding, medical imagery did not hold true knowledge when intertwined with differing voices from patients’ experiences of pain. Such diagnostic tools also contributed to increasing the understanding of hand OA in consultations. Results that aligned with patients’ symptoms facilitated stronger involvement of patients in the dialogical encounter, thereby providing patients with legitimacy to bring their own experiences more actively out of the periphery and towards the centre of the dialogue.

Patient experiences were also relevant epistemic resources in decisions regarding intra-articular injections. Although, in interviews and during observations, health professionals pointed to how voices from medical research and international recommendations in rheumatology did not advise such practices, some health professionals would still provide patients with injections: “*I have a lot of patients who have positive experiences from before. They [patients] feel that it [injection] has helped so much in the past that it’s difficult to argue against, even if there is perhaps not so much inflammation. Then there is the fact that the patient has had positive experiences of it [injection] in the past, which probably makes us continue to offer it [the injection]*” (R5). This illustrates how the experiences of patients in concert with the voices of health professional knowledge in local contexts create a dialogue of equals. Similar dialogues took place when patients reported inactivity as a response to hand pain when they understood pain as representing a warning of danger. Health professionals conversely encouraged patients to carry out regular activities of daily life and to do hand exercises despite pain, informing patients that pain during activity was normal and harmless, and could even contribute to pain reduction. In such encounters, knowledge about hand OA to inform practice was not imposed by health professionals as a monologue but was rather constructed through a dialogical exchange of ideas. This shows how different voices in dialogue are important to the process of understanding and have consequences for the negotiations and the actions taken in managing and coping with hand OA.

Within our study context, where health professional knowledge was often taken for granted, patients also described encounters with health professionals who voiced that they did not know the solution to patient problems: “‘*I know so little about it’, he said. ‘So, if you’re told that there’s help out there for this, just let me know and I’ll refer you there.’ And that’s how he was with my fingers too—he said, ‘I’ll give you a referral, as I have no idea about this’ […] Yes, he almost admits that he doesn’t know much about it. ‘I’ll pass you on.’ And that’s the right thing to do*” (P13). In this situation, the patient did not talk about the health professional’s not knowing how to take action as a negative attribute, rather she approved of his ability to acknowledge his limitations. This resonates with health professionals in our study who openly shared with patients that they did not know: “*I’m very careful about not answering things I don’t know about. Then it’s better to say that I don’t know*” (OT7). Such situations of health professionals not knowing also occurred in the consultations we observed, where clinical uncertainties were shared with patients. In one consultation where a man in his forties with an IT background presented hand pain to a rheumatologist, the rheumatologist initially pointed to his symptoms as differing from hand OA. She included the patient in her clinical reasoning of doubt and involved him in the process of reducing diagnostic uncertainty. She openly engaged in reasoning about whether this was hand OA or another condition. On several occasions, she emphasized that she did not know and said to the patient that she should have prepared more in advance by reading about conditions outside of her own field of expertise. In the encounter, she accessed online resources, initially with some difficulties, and used Google and UpToDate (an evidence-based clinical decision-support resource) to complement her own resources, thereby bringing digital scientific voices into the dialogue. When identifying what she found to be relevant information, she read sentences aloud from the computer screen in English, directly followed by a Norwegian translation. The information from UpToDate corresponded with her initial understanding of his hand symptoms as not being related to hand OA. By voicing ambiguities with the patient, the rheumatologist brought her own position of not knowing to the centre of the dialogue. Involving the patient in her clinical reasoning when she did not know the answers to his challenges enabled them to collectively make sense of the patient’s hand pain.

### Dialogue takes place from hybrid positions when patients are health professionals and health professionals have hand OA

Our final theme captured the construction and negotiation of hand OA from hybrid positions when patients had health professional backgrounds, and when health professionals lived with hand OA. Although such a polyphony of voices was presented within one voice and was made use of in different ways in clinical encounters, study participants in hybrid positions blended their illness experiences and professional knowledge within one voice to make sense of hand OA. Patients in our study with a health professional background reported that they did not openly disclose their status as health professionals in consultations with rheumatologists and occupational therapists: “*I don’t express it [health professional background] myself […] then they [the other health professionals] must have read my papers [medical records]*” (P4). “*I don’t tell the doctor much, or I don’t say what I do for a living*” (P7). By positioning themselves openly as patients only in the external dialogue with health professionals, they simultaneously silenced their own knowledge from being health professionals in the dialogical encounter.

Concurrently, these participants talked about the added value of having the ability to “*see things from the other side*” (P12), bringing the health professional’s voice closer to the centre of the inner dialogue, using resources from being a health professional as an asset that provided directions for action in dialogue, even though it was not openly disclosed in consultations. They also pointed to a more privileged position compared to other patients: “*I feel sorry for those who don’t have it [health professional background] actually, in many circumstances. That’s why I have to say that I’m glad I have that foundation, as a health professional. Yes, I really am glad, yes, and it provides great security, for myself*” (P8). This shows how the experiences from also being health professionals impacted how patients positioned themselves when they made efforts to act in accordance with what they thought of as being good patients: “*[…] then I felt maybe that I was a bit of a difficult patient again, right, because I don’t want to be a difficult patient as I myself have dealt with difficult patients for many years. So, I didn’t feel like it [being a difficult patient]*” (P14). Thus, the health professional background of patients shaped the illness presentation in consultations. The patient’s previous experiences with difficult patients, in the role as a health professional, were brought into the encounter in such a way that she adjusted her positioning to align with the expectations of the health professional she was interacting with. This shows how a multitude of voices within and between the actors play out in the clinical encounter when patients are also health professionals.

Health professionals who had experiences of living with hand OA reported that they openly communicated that they also had hand OA in interactions with patients: “*I have hand osteoarthritis myself, so I often use myself as an example. I consider the patient, of course, but I often find it meaningful to say that I have the medical knowledge that is hopefully required, but that I also have personal experience in this field […] it doesn’t always feel natural to bring oneself into the conversation, but sometimes I do it successfully, because I think it’s nice to share experiences in terms of what kind of NSAIDs you can use and the dosages, about exercises, yes, those kinds of experiences*” (R3). Such situations were further observed in consultations where health professionals made comparisons between the hands of patients and their own hands when explaining hand OA. By bringing their own experiences of having hand OA to the fore in clinical encounters, the relevance of such experiences became central in the construction of meaning. Consequently, the health professionals’ subjective experiences of hand OA contributed to enhancing the collective understanding of hand OA in a dialogue between interactants with similar experiences. In this way, the actors were positioned to identify with each other without questioning the other’s ability to understand. This interconnectedness not only facilitated negotiations from more equal positions but also contributed to an increase in the value of personal experiences in the understanding and management of hand OA.

## Discussion

Contemporary healthcare is informed by many different sources of knowledge (Rycroft-Malone et al., 2004) that vary in status and thus in how strongly they are voiced. Two paradigms demonstrate this multiplicity: increasingly standardized and evidence-based healthcare versus participation and patient-centred healthcare. The construction of ideas about hand OA takes place at their intersection. This context presents contested relations and asymmetries in power, knowledge and practice that are brought into clinical encounters and that determine which voices are valued and which are not. Each consultation is full of paradoxes regarding what constitutes true knowledge, illustrating the complexity of healthcare and the multitude of voices that people act from and towards in interactions (Barry et al., 2001).

The taking for granted of what constitutes valid and relevant knowledge in healthcare contributes to the historical and cultural stability of interactions where health professionals dominate how knowledge is constructed in the clinical encounter (Pilnick & Dingwall, 2011; Rose & Kalathil, 2019). Through such providers-know-best practices (Bontemps-Hommen et al., 2020; Joseph-Williams et al., 2014), voices of patients are silenced (Prytz Mjølstad et al., 2013), as exemplified in our first theme. Patients are conditioned to understand their own voices as irrelevant. This endorsement of a single, dominant health professional voice is framed within the current evidence-based healthcare paradigm, where the best available evidence becomes the central source of knowledge (Timmermans & Mauck, 2005). This in turn regulates and sustains an agreement that health professionals know best, thereby limiting alternative voices. When voices of health professionals dominate dialogue, knowledge about hand OA is constructed as an entity that belong to health professionals who actively transfer that knowledge to patients in the periphery in need of that expertise (Dingwall & Pilnick, 2020). Such constructions are driven by science and the accrued clinical experiences of health professionals, which creates a steady division between healthcare realities and the lifeworld of patients in harmonious relationships (Parsons, 1951).

However, the ways in which hand OA is understood and managed is not a truth that applies always and everywhere. If knowledge from science were to be regarded as the given truth for clinical practice and gold standards rigidly followed, knowledge about chronic illness within practice would not be considered (Gergen, 1978, 1994b). Chronic illness and the absence of a cure contribute to transforming the dialogue about what constitutes valid knowledge in healthcare (Pilnick, 2022; Strauss, 1985), shifting the focus from what health professionals know to how to manage everyday life with chronic illness, as shown in our second theme. Centrifugal forces are evident when health professionals and patients voice opposing ideas about experience as knowledge in the chronic illness dialogue. In parallel, centripetal forces towards balance and unity are brought into being through patient-centred and participatory approaches that frame contemporary healthcare and actions in consultations. Knowledge is never a static individual asset, but is relational in nature (Gergen, 2015), where voices from patient experiences increase in strength in the construction of valid knowledge about hand OA (Szasz & Hollender, 1956), recognizing patients as knowers in healthcare (Byrne & Long, 1976). Thus, the dialogue between health professional knowledge from science and the understanding of illness through patient experiences is changed, moving the former closer to the periphery while positioning the latter closer to the centre of the conversation (Freidson, 1970).

When constructing new knowledge spaces, opportunities to voice shared decisions, partnerships and patient-centred practice increase (Mead & Bower, 2000) as interactants participate from more equal positions of influence (Gibson et al., 2012; Jasanoff, 2004). This is consistent with our third theme. In encounters where health professionals and patients co-construct knowledge, centripetal and centrifugal forces are continuously in play to create a balance. The health professionals’ orientation towards general, objective and relevant ideas about disease from biomedical sources opposes patients’ particular, subjective and real illness experiences. At the same time, centripetal forces towards unity of meaning function through shared objectives to understand and address individual patient needs in singular consultations. In chronic illness trajectories that do not present with a start and an end, the construction of knowledge becomes an enduring process through dialogue. It becomes an ongoing task for patients and health professionals in concert, sometimes dancing and at other times duelling with each other in the clinical encounter (Stivers & McCabe, 2021). As a result, both parties endeavour to share understandings that exceed the sounds of individual voices through egalitarian and collaborative dynamics (Sainsaulieu, 2023).

Furthermore, in our study, the clinical communication of uncertainty by health professionals, a phenomenon that is incompletely understood (Han et al., 2019), is worth noticing. Our analysis showed how claims of not knowing the answers to patient problems were made openly in consultations. A study by Tai-Seale et al. (2012) focused on how epistemic uncertainty is seldom communicated in clinical practice, and when it is communicated may reduce patient trust and satisfaction (Politi et al., 2011). However, we would argue that the expression of uncertainty by health professionals contributed to enhancing the dialogue in the formation of new knowledge. Prior research reinforces our view that claims of not knowing contribute to building patient-provider partnership (Gordon et al., 2000), thus facilitating knowledge co-development within the context of its use.

Finally, the vantage point of being patients with a health professional background or health professionals with hand OA reflected the multiplicity of voices within one voice in the knowledge construction process. These in turn reinforced the collective quality of ideas from centrifugal forces of opposition. Patients with a health professional background were reluctant to openly disclose their professional status in consultations, which is in line with a study by Ingstad and Christie (2001). Although they did not use their health professional voice openly in dialogue with other providers, they made efforts to be good patients (Stanton & Randal, 2016) when they used their health professional voices from within. This inner voice was additionally used to make sense of hand OA and the accompanying healthcare services in the consultation dialogue. Conversely, when health professionals made their own hand OA diagnosis central in the clinical encounter, epistemic connections could be formed through shared and embodied knowledge (Battalova et al., 2020). Centripetal forces bring balance when meaning is unified through interactants’ sharing of ideologies from both experiential sources and from professional knowledge, which contributes to a unifying language in dialogue. This therapeutic use of self (Solman & Clouston, 2016; Taylor et al., 2009) contributed to optimizing the dialogue with patients. These voices from professionals and patients were orchestrated in combination with numerous other voices from within, including age and gender. This polyphony balanced unifying and dividing forces to create a dialogue between the consultation interactants.

Our study of how hand OA meaning-making is talked into being in patient-provider encounters epitomizes the complexity and fluidity of who holds legitimate knowledge about hand OA. Our analysis shows how knowledge is voiced and exercised variously, encounter by encounter, through balancing the centripetal forces towards unity and the centrifugal forces from opposing ideas. In conclusion, the combination of health professional ideas and patient experiences from a plurality of knowledge sources creates a myriad of polyphonic processes in local contexts, when dialogue paves the way for new knowledge to be understood in its co-creation (Gergen, 2011).

This study comes with limitations that need to be acknowledged. Although we communicated our commitment to participant confidentiality in enabling a safe environment and free speech, we acknowledge that dominant Norwegian policies on patient-centred care and evidence-based healthcare influenced what it was possible for health professionals and patients to talk about and do when they represented their workplace, their profession and themselves in a complex interweaving of historical, cultural and societal voices that were active in the clinical and interview dialogue. We have therefore limited reporting of participant demographics (Morse & Coulehan, 2014). HJM was the only research team member immersed in the field, and by engaging with co-authors and peers, efforts were made to balance closeness to the field with an analytical distance throughout the research process. As HJM is not a trained ethnographic observer, preparatory work prior to data generation in a rheumatology hospital not included in this study took place in order to practice ethnographic skills in efforts to move observations from ordinary and clinical looking to systematically and rigorously observing (Patton, 2002).

## Conclusion

Our study sought to explore contemporary construction of knowledge about a chronic condition of the hands, and how patients and health professionals were situated in relation to understanding hand OA. Through our analysis, we have shown that knowledge is not a fixed property that can be held by health professionals or patients in clinical encounters. From a dialogical perspective, co-constructed knowledge about hand OA originates in the flexible and fluid character of the interactants’ expertise in interaction, including illness experiences. Knowledge about a chronic health condition as such is situated and co-constructed in the context of the clinical encounter through the multiplicity of voices, dominant and peripheral, within and between the interactants in dialogue.

## Supplementary Material

Supplementary material 1 Interview guides.docx

## Data Availability

Data not available due to ethical restrictions.
