# Supplementary material for: Dialogued into being: Constructing knowledge about hand osteoarthritis from a polyphony of voices in healthcare encounters
Source: Int J Qual Stud Health Well-being. 2024 Mar 18;19(1):2330221. doi: 10.1080/17482631.2024.2330221 (PMC10949837; doi:10.1080/17482631.2024.2330221)
Supplement: Supplementary material 1 Interview guides.docx [file ZQHW_A_2330221_SM7827.docx]

**Supplementary material 1. Interview guides A and B**

1. **Interview guide persons with hand OA**

***Symptom manifestation, initial contact with healthcare services, and diagnosis***

- Examples questions:
  - How did you notice the symptoms in your hands?
  - What did you do about it?
  - What were your reasons for contacting healthcare services?
  - What happened when you consulted healthcare services?
  - How were you informed about having hand OA?
  - What did you know about hand OA prior to the healthcare consultations?
  - Where did that information come from?
  - What was important for you in seeking healthcare for your hand OA?

***In consultations***

- Examples questions:
- How would you describe the consultations you had with rheumatologists/occupational therapists/general practitioners?
- Can you describe what happened during consultations?
- What information did you get regarding your hand OA?
- How did the consultations match your expectations?
- In what ways were you listened to regarding your hand OA condition?

***After consulting healthcare***

- Examples questions:
  - What healthcare services where you provided?
  - What were your experiences with those healthcare services?
  - How do you understand hand OA after being in contact with healthcare?

1. **Interview guide health professionals**

***In consultations with persons with hand ailment***

- Examples questions:
  - How would you describe a typical consultation with a patient with hand ailment?
  - What types of challenges/concerns do hand OA patients present to you?
  - How do you respond to those concerns?
  - How do you respond to patient experiences and ideas?
  - How does patient ideas about hand OA correspond with your professional understandings?

***Knowledge and experience***

- Examples questions:
  - Where are the insights/skills you have about hand OA originating from?
  - What insights/skills do you find important in addressing the needs of persons with hand OA?
  - What professional tasks do you have in encounters with persons with hand ailment?
  - How do you use those professional tasks in consultations?
  - How do you stay updated on new developments regarding hand OA?
